# Identification of a novel peptide ligand for the cancer-specific receptor mutation EGFRvIII using high-throughput sequencing of phage-selected peptides

**DOI:** 10.1038/s41598-022-25257-4

**Published:** 2022-12-01

**Authors:** Sourour Mansour, Indranil Adhya, Coralie Lebleu, Rama Dumpati, Ahmed Rehan, Santu Chall, Jingqi Dai, Gauthier Errasti, Thomas Delacroix, Raj Chakrabarti

**Affiliations:** 1 Center for Protein Engineering and Drug Discovery, PMC Isochem SAS, 32, rue Lavoisier 91710, Vert-Le-Petit, France; 2 Division of Computational Research, Chakrabarti Advanced Technology, Hyderabad, Telangana India; 3 Chakrabarti Advanced Technology, LLC, PMC Group Building, 1288 Route 73, Ste 110, Mount Laurel, NJ 08054 USA

**Keywords:** Cancer, Computational biology and bioinformatics, Drug discovery

## Abstract

We report here the selection and characterization of a novel peptide ligand using phage display targeted against the cancer-specific epidermal growth factor tyrosine kinase receptor mutation variant III (EGFRvIII). This receptor is expressed in several kinds of cancer: ovarian cancer, breast cancer and glioblastoma, but not in normal tissues. A 12-mer random peptide library was screened against EGFRvIII. Phage-selected peptides were sequenced in high-throughput by next generation sequencing (NGS), and their diversity was studied to identify highly abundant clones expected to bind with the highest affinities to EGFRvIII. The enriched peptides were characterized and their binding capacity towards stable cell lines expressing EGFRvIII, EGFR wild type (EGFR WT), or a low endogenous level of EGFR WT was confirmed by flow cytometry analysis. The best peptide candidate, VLGREEWSTSYW, was synthesized, and its binding specificity towards EGFRvIII was validated in vitro. Additionally, computational docking analysis suggested that the identified peptide binds selectively to EGFRvIII. The novel VLGREEWSTSYW peptide is thus a promising EGFRvIII-targeting agent for future applications in cancer diagnosis and therapy.

## Introduction

One of the few known cancer-specific cell surface markers, is the epidermal growth factor (EGF) tyrosine kinase receptor mutation variant III (EGFRvIII)^1^. The mutated receptor lacks amino acids residues 6–273 in the extracellular domain compared with the wild-type EGF receptor (EGFR WT), and deletion of those 268 amino acids creates a junction site with a new glycine residue between amino acids 5 and 274^2^. EGFRvIII does not contain a ligand-binding domain and is constitutively active^3,4^.

Although the kinase activity of EGFRvIII is much weaker than for ligand-activated EGFR WT, it was found sufficient to confer a growth advantage to tumors^5,6^. EGFRvIII is present in a number of human malignancies such as glioblastoma, lung cancer, and breast cancer^7^. EGFRvIII is the most common EGFR mutation occurring in patients with glioblastoma, being expressed in 20–50% of glioblastomas^8–10^. Prevalence is the highest in patients with tumors overexpressing EGFR WT^9^, and EGFRvIII is considered to occur after amplification of EGFR WT^11^. However, in contrast to EGFR WT, which is expressed in most mouse and human healthy cells, EGFRvIII was never identified in normal tissues. Thus, EGFRvIII can be used as a target for cancer diagnosis and therapy, especially of glioblastomas.

Tumor-targeting ligands such as peptides and antibodies may effectively aid in delivery of certain cytotoxic agents (either biological or synthetic) to the tumor cells, thereby improving therapeutic efficacy while limiting the exposure of normal tissues to the cytotoxic agents^12^. Therapeutic approaches have included the use of unarmed monoclonal antibodies (MAbs)^13,14^ radiolabeled MAbs^15^, MAbs conjugated to immunotoxins^16,17^, or boronated dendrimers^18^. A single-chain variable fragment (scFv) specific to EGFRvIII was discovered and integrated in a tandem antibody (TandAb) construct^19^. Small peptides (< 5 kDa) that selectively recognize tumor cells have advantages over MAbs (150 kDa), TandAbs (100 kDa) and scFvs (20–30 kDa) since they are easy to synthesize and modify due to their much lower size, have higher cell membrane penetration, and possess less immunogenicity^20^. Even if the binding affinity of peptides is lower compared to antibodies and fragments, avidity can be increased by incorporating multiple copies of peptides on the surface of nanostructures when developing tumor-targeting delivery systems^20^.

Regarding tumor imaging applications, to date, only 12 cancer mutation-specific tracers have been approved by the FDA, with none for brain tumors. Peptide tracers are preferred to antibody tracers due to their low immunogenicity and low cost^21^, as already stated above, but also for their rapid clearance from blood and non-target tissues, their shorter injection-to-imaging time and because they are compatible with shorter-lived isotopes^22,23^. Thus, there is a need to develop new peptides capable of binding with high specificity to EGFRvIII compared to EGFR WT for diagnostic, therapeutic or theragnostic applications—as imaging agents using radiolabeling or fluorophore labeling^21,22^, in peptide-drug conjugates^24^, or in targeted delivery systems^20,21,25^.

Two short peptides intended for EGFR targeting of tumor cells have been described in the prior art. The FALGEA peptide is described as binding to both EGFR WT and EGFRvIII^26–28^. The YHWYGYTPENVI peptide^29^, discovered from the GE11 peptide^30,31^, has been proven to bind to EGFR WT, while its potential binding to EGFRvIII is unknown.

The aim of this study was to identify a novel peptide binding to EGFRvIII using phage display, a widely used method for the development of peptide ligands. In our strategy, the procedure involved three iterative rounds of affinity selection and phage amplification followed by a high-throughput sequencing (HTS) to analyze the abundance and diversity of isolated peptides. The binding of the screened phage-displayed peptides to the protein target EGFRvIII was tested on cells and characterized by flow cytometry. Only potential hits were synthesized to confirm their cellular binding, and their binding affinity was assessed by microscale thermophoresis (MST) and computational docking.

## Results

### Enrichment of EGFRvIII-binding peptide-phages

Three rounds of affinity selection were performed to select specific EGFRvIII-binding phages from the Ph.D.™-12 Phage Display Peptide Library using purified and active extracellular domain of the EGFRvIII protein. Quantitative polymerase chain reaction (qPCR) was used to accurately enumerate phage particles that bind to the substrate and determine the phage enrichment with each round over the background (Fig. S1). High phage enrichment rate was observed after each round, which indicates successful affinity selection of panned phages for the EGFRvIII target.

### Diversity of phage-selected peptides

**Figure 1:**
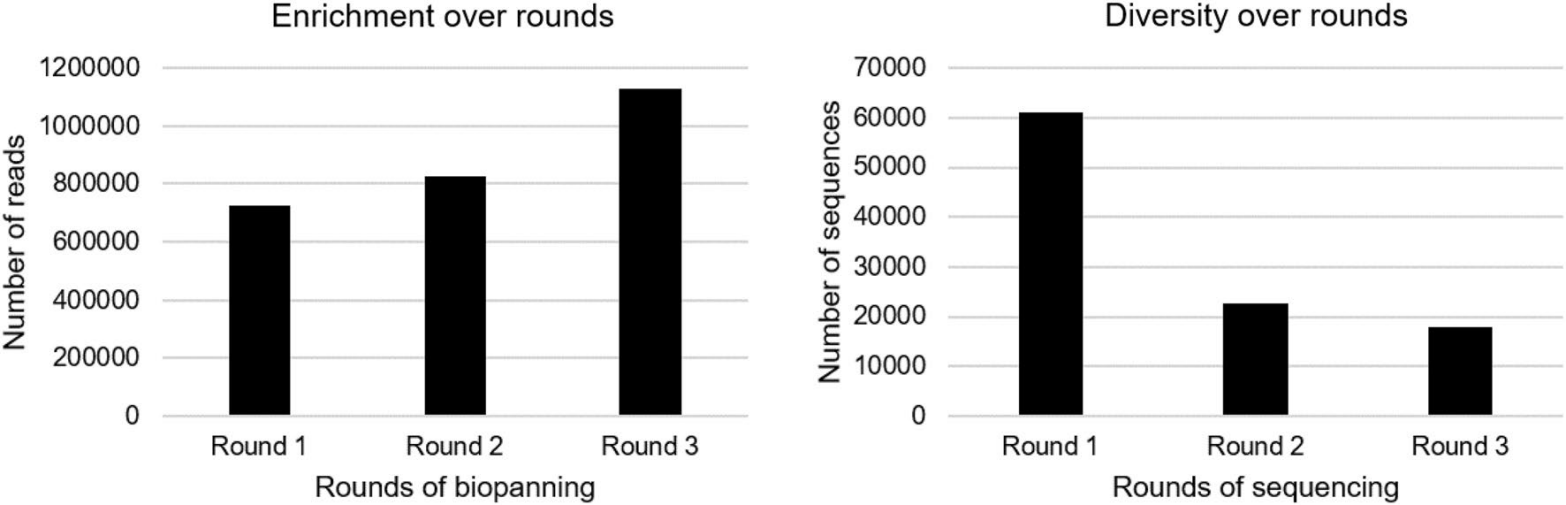
The total number of reads and sequences in each round of biopanning. The Y-axis represents the number of reads (left) or sequences (right). The X-axis represents each round of biopanning.

The eluates from the three rounds of biopanning and the background were sequenced by Illumina following PCR with the adaptor and the next generation sequencing (NGS) data was analyzed. Results show that the total number of reads increased over the rounds while the diversity of the peptides decreased (Fig. 1), meaning that EGFRvIII-specific peptides were recovered.

### Evaluation of the specific binding of the phage-selected peptides

**Figure 2:**
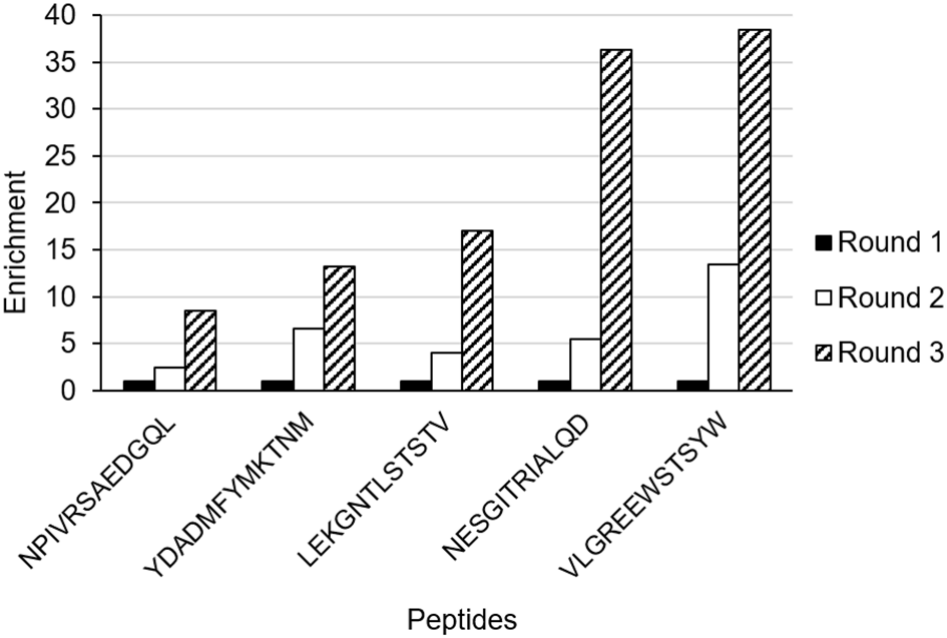
The top peptides enrichment in each round of biopanning. The Y-axis represents the enrichment and the X-axis represents the peptide sequences.

Five top peptides were selected from the NGS data analysis: YDADMFYMKTNM, LEKGNTLSTSTV, VLGREEWSTSYW, NPIVRSAEDGQL and NESGITRIALQD (Fig. 2). The criteria used for the selection was that the peptides should enrich over the rounds of biopanning and, if present in the background, the frequency of these peptides should be below 0.002.

The binding specificity of these five phage-displayed peptides was evaluated directly with phages on stable cell lines expressing EGFRvIII and EGFR WT followed by flow cytometry characterization. The parental cell line, 293T, was used as a negative control as it expresses very low levels of endogenous EGFR WT, does not express EGFRvIII and is a non-cancerous cell line.

**Figure 3:**
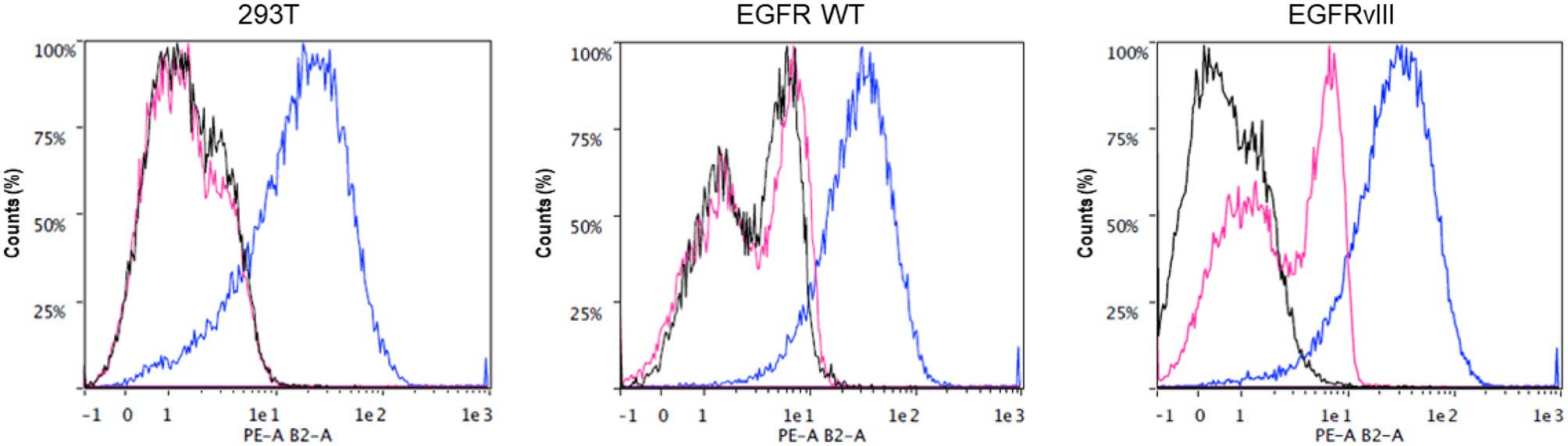
VLGREEWSTSYW-M13 binding analysis by flow cytometry. 293T, EGFR WT and EGFRvIII cell lines were incubated with the VLGREEWSTSYW-M13 phage-displayed peptide. Cell counts in % versus PE signal: when incubated with primary + secondary antibody (anti-M13_biotin + anti-biotin_PE, pink), isotype labelling + secondary antibody (anti-IgG3_biotin + anti-biotin_PE, blue) or secondary-only antibody (anti-biotin_PE, black).

Among the tested phage-displayed peptides, only VLGREEWSTSYW-M13 showed a specific binding to EGFRvIII, giving confidence that the phage was binding to the cell-surface exposed region of its respective target antigen. Indeed, the fluorescence intensity of VLGREEWSTSYW-M13 for the EGFRvIII cells was stronger when cells were treated with the primary antibody (anti-M13) compared to the secondary-only antibody control, while there was no significant difference in these fluorescence intensities for the EGFR WT cells and the 293T cells (Fig. 3). In contrast, the other tested phage-displayed peptides such as NPIVRSAEDGQL and NESGITRIALQD did not show a specific binding (Fig. S2). The isotype control antibody was used in this phage-cell surface staining protocol to rule out non-specific Fc receptor binding of the anti-M13 primary antibody. The same antibody concentration for both the isotype control and the primary antibody was used in the experiment. The isotype control antibody did not perform as expected and showed high levels of non-specific fluorescence. Since the exact same levels of non-specific fluorescence were found in all the three cell lines with the isotype antibody in the experiment without phage (Fig. S3) and with phage (Fig. 3), we estimated that the difference of binding observed with the anti-M13 antibody on the EGFRvIII cell line is specific and not due to a background staining.

### Validation of the specific binding of the VLGREEWSTSYW peptide

To confirm the results, a synthetic VLGREEWSTSYW peptide bound to a fluorescent marker was synthesized and the binding specificity towards EGFRvIII versus EGFR WT was evaluated in vitro. To this end, the VLGREEWSTSYW peptide was synthesized and conjugated with a fluorescent marker fluorescein isothiocyanate (FITC) at the N-terminus using a 6-aminohexanoic acid (Ahx) as a linker. Two peptides from the literature were also synthesized with this FITC-Ahx tag to serve as controls. The FALGEA peptide should be able to target both EGFR WT and EGFRvIII^19–21^, while the YHWYGYTPENVI peptide should only bind to EGFR WT^22^.

**Figure 4:**
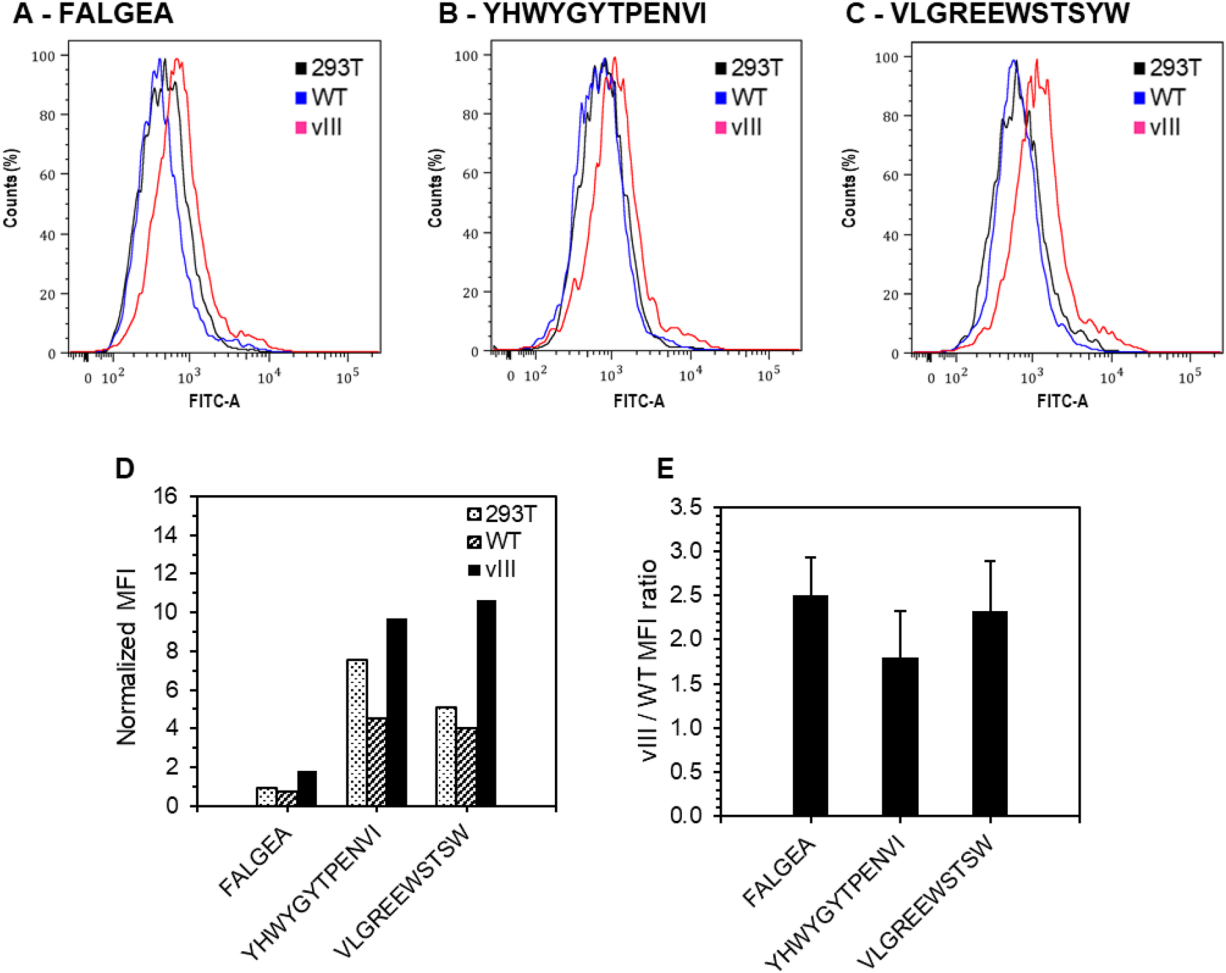
Quantitative cellular binding of FITC-Ahx-peptides on 293T, WT and vIII cells by flow cytometry. (**A–C**) 293T cells (black), EGFR WT cells (blue) and EGFRvIII cells (red) were incubated with 10 µM of FITC-Ahx-peptides: (**A**) FALGEA, (**B**) YHWYGYTPENVI, (**C**) VLGREEWSTSYW for 20 min at RT. (**A–C**) Cell counts in % versus FITC signal. (**D**) Normalized mean fluorescence intensities (MFI) of each peptide for each cell line. (**E**) Ratio (mean and standard deviation) for each peptide of the normalized MFI obtained for EGFRvIII cell line over EGFR WT cell line at 3 different concentrations 1 µM, 5 µM and 10 µM.

The 293T, EGFR WT and EGFRvIII cell lines were incubated with 1, 5 and 10 µM of the three FITC-Ahx-peptides for 20 min at RT (Fig. S4). FITC fluorescence intensity of the cells was measured by flow cytometry. Peaks and mean fluorescence intensities (MFI) were determined for 10 µM and the results are shown in Fig. 4 and Table 1. A shift of fluorescence was observed for the VLGREEWSTSYW peptide on EGFRvIII compared to 293T and EGFR WT cell lines (Fig. 4C). This shift was higher than for the control peptides (Fig. 4A,B).

**Table 1:** Normalized MFI values and vIII/WT ratios for each peptide and each cell line obtained by flow cytometry.

Sequence	Normalized MFI at 10 µM			Normalized MFI ratio vIII/WT from 3 different concentrations	
	293 T	WT	vIII	Mean	SD
FITC-Ahx-FALGEA-CONH_2_	0.9	0.7	1.8	2.50	0.44
FITC-Ahx-YHWYGYTPENVI-CONH_2_	7.6	4.5	9.7	1.80	0.52
FITC-Ahx-VLGREEWSTSYW-CONH_2_	5.1	4.0	10.7	2.33	0.56

Normalized MFI data show that the binding to EGFRvIII cells in the tested conditions was much higher for VLGREEWSTSYW compared to the FALGEA control (Fig. 4D). Furthermore, the non-specific interactions (293T cell binding) were minimized for VLGREEWSTSYW compared to the YHWYGYTPENVI control.

Finally, to assess the EGFRvIII-binding specificity, the ratio of MFI between EGFRvIII and EGFR WT was calculated for the three peptide concentrations and shows the specificity to EGFRvIII versus EGFR WT for VLGREEWSTSYW compared to the control peptide YHWYGYTPENVI (Fig. 4E).

Altogether the data show that the VLGREEWSTSYW peptide binds to EGFRvIII cells with high efficiency and good specificity compared to EGFR WT cells and with less nonspecific interaction (293T cells). In conclusion, VLGREEWSTSYW is a better targeting peptide for EGFRvIII-expressing cancer cells than peptides known from the art.

### Peptide binding affinity

The binding affinity of the peptides to EGFRvIII and EGFR WT in solution phase was first evaluated by microscale thermophoresis (MST) (Fig. S5). Solution phase Kd’s were measured to validate binding and selectivity rather than to quantify binding affinity on the cell surface, due to the differences in the conformational epitope for activated EGFRvIII displayed on the cell surface. For both peptides, the MST curves of EGFRvIII (right panel) show an increased signal, whereas the ones of EGFR WT (left panel) are flat. These observations indicate that VLGREEWSTSYW and FALGEA preferentially bind to EGFRvIII rather than EGFR WT.

**Figure 5:**
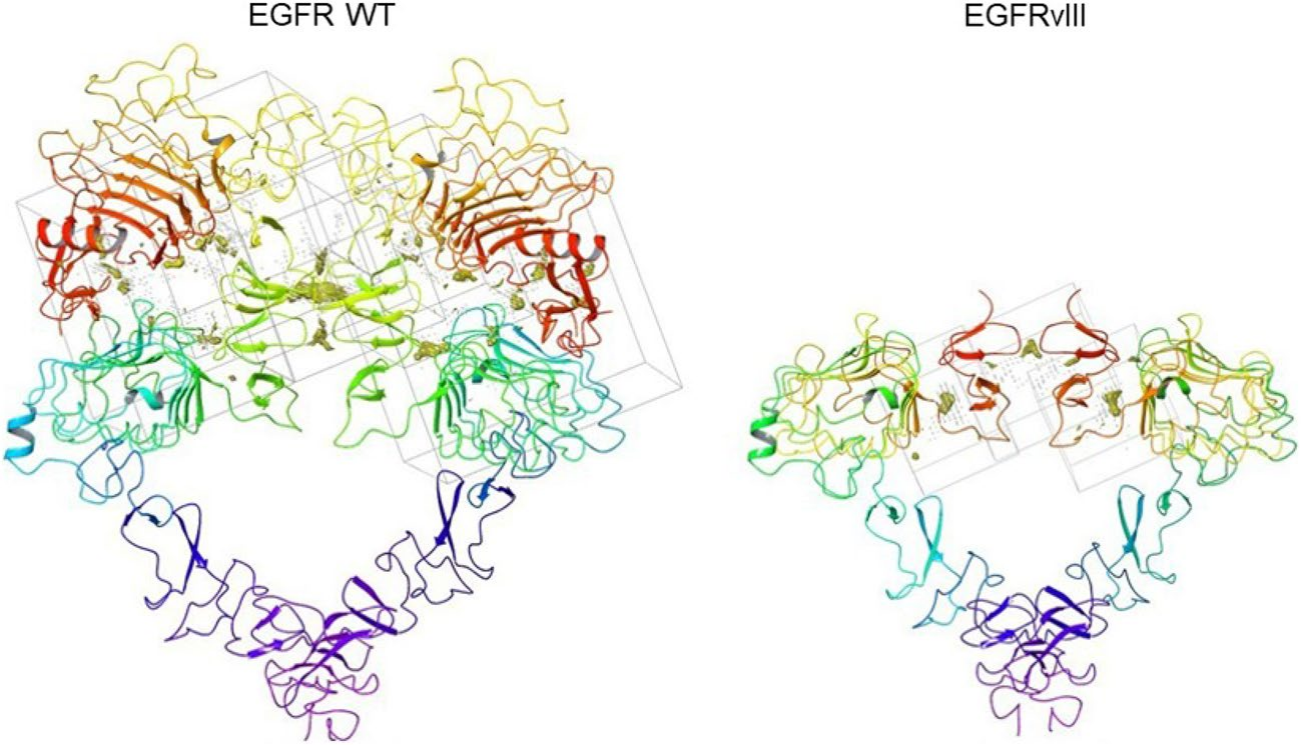
Structures and active site regions of EGFR WT and EGFRvIII proteins. The figure shows EGFR WT with 1388.2 cubic Å hydrophobic region (left) and EGFRvIII with 183.4 cubic Å hydrophobic region (right). The cubes show the secondary structures of both proteins.

We then simulated the peptide affinity by computational docking, a common and robust approach to predict protein–protein/peptide interactions that expedites the peptide discovery cycle. Various parameters such as binding affinity, specific residues involved in the interaction and number of stable conformations can be obtained rapidly. These data are useful as a basis for peptide screening to be further exploited for experimental validation. Since the 3D model of EGFRvIII was not available, the protein structure was obtained by homology modelling. Modelling and subsequent energy minimization resulted in the conformation used for active site search. The 6–273 amino acids in the EGFR WT protein were removed from both chains, and the cross connected disulfide bridges were reduced in number. The structure was stable by forming two disulfide interactions in each chain from Lys 304 to Asp 279. The minimization protocol of 100 steepest descent steps at 0.02 Å cut-off, followed by 10 conjugate gradient steps with the same cut-off range resulted in a local minimum structure which had a root-mean-square deviation (RMSD) of 0.9 Å. The binding sites in the protein were explored using CastP server tool, based on theoretical and algorithmic results of the computational geometry of the proteins. Five hydrophobic pockets were found present in the dimer, towards the N-terminal. The structures and active site regions of both EGFR WT and EGFRvIII are represented in Fig. 5.

Docking was carried out using HPEPDOCK server tool by considering the active sites as a search space. The docking scores are represented in Table 2. Peptides presenting a good binding affinity and the ability to form a maximum number of complexes by interacting with any of the active sites of the EGFRvIII protein were considered as putative hits. The results show that the VLGREEWSTSYW peptide has a lower docking score than the other peptides selected by NGS including LEKGNTLSTSTV, NPIVRSAEDGQL and NESGITRIALQD. This confirms that VLGREEWSTSYW has a good affinity for EGFRvIII.

**Table 2:** Peptide docking results on EGFRvIII: docking scores and number of stable conformations that the peptide can form with the active site regions of the EGFRvIII protein.

Peptide Sequence	Docking score (kcal/mol)	Number of stable conformations
VLGREEWSTSYW	−186.5	23
LEKGNTLSTSTV	−140.5	25
NPIVRSAEDGQL	−148.7	38
NESGITRIALQD	−150.0	13
YDADMFYMKTNM	No interaction	7

### Characterization of interactions between VLGREEWSTSYW and EGFRvIII by computational docking

**Figure 6:**
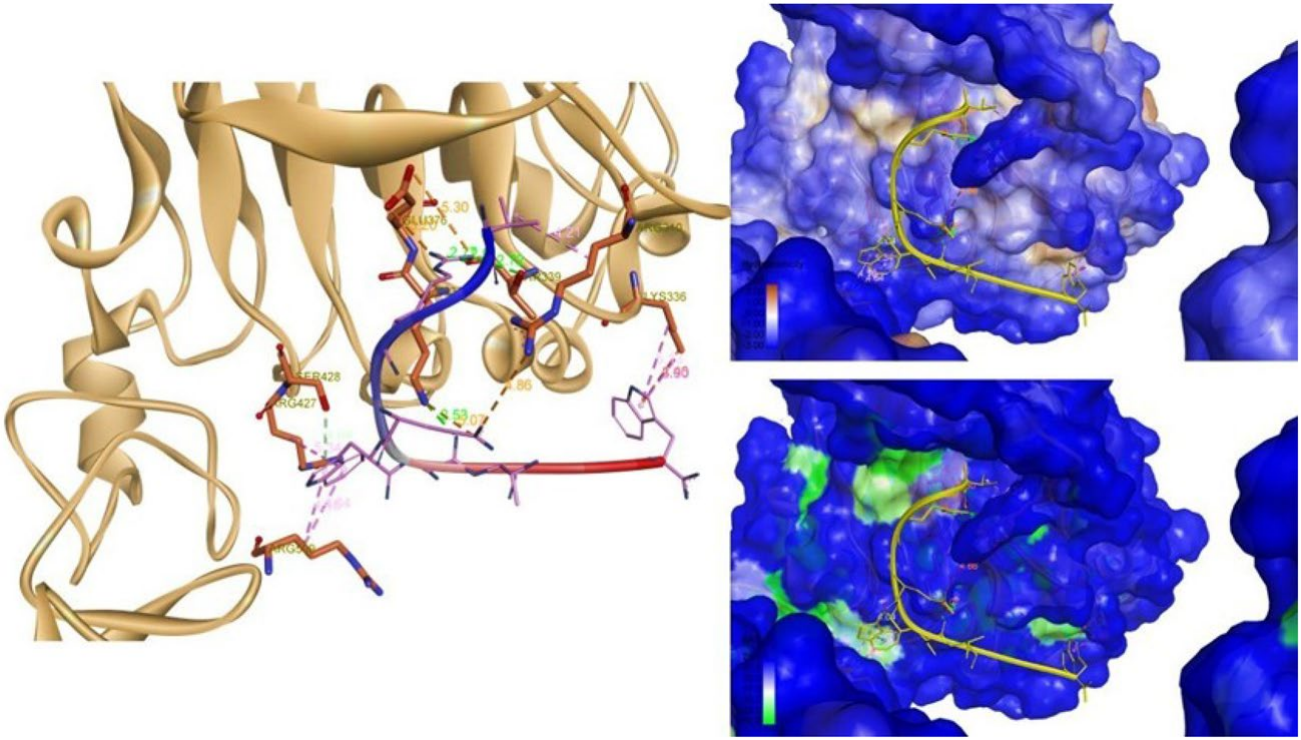
Inter-molecular interactions in the EGFRvIII–VLGREEWSTSYW complex. The peptide is strongly bound to the protein using Arg 4 to Thr 339. Trp 7 binds to Ser 428 and, Ser 8 to Lys 375. There are several hydrophobic interactions holding the peptide to the hydrophobic pockets on both ends of the peptide.

The docking study of the VLGREEWSTSYW peptide with the EGFRvIII showed several inter-molecular interactions in the protein-peptide complex (Fig. 6 and Table 3). Hydrogen bonds are formed with the residues Lys 375, Thr 339 of EGFRvIII, a π-donor hydrogen bond with Ser 428 and a π–σ bond with Lys 336. The Trp 7 and 12 of the peptide form a π-alkyl interactions with Arg 509, Arg 427, Arg 509 and Lys 336. The Val 1 and the Trp 12 of the peptide form hydrophobic bonds respectively with Arg 310, Lys 336. The peptide is decently accommodated in the pocket with no solvent accessibility resulting in a stable complex. Altogether, these results indicate that the peptide strongly holds in the hydrophobic cavity of EGFRvIII.

**Table 3:** Inter-molecular interactions between EGFRvIII and VLGREEWSTSYW: amino acids involved, distance and type of interaction.

Number	From	To	Distance (Å)	Type
1	ARG 310	GLU 6	4.863	Electrostatic
2	LYS 375	GLU 6	5.073	Electrostatic
3	ARG 4	GLU 376	5.304	Electrostatic
4	ARG 4	GLU 376	3.197	Electrostatic
5	LYS 375	SER 8	2.527	Hydrogen bond
6	ARG 4	THR 339	2.914	Hydrogen bond
7	ARG 4	THR 339	2.719	Hydrogen bond
8	ARG 4	THR 339	2.774	Hydrogen bond
9	LYS 336	TRP 12	4.450	Electrostatic
10	SER 428	TRP 7	4.041	Hydrogen bond
11	LYS 336	TRP 12	3.904	Hydrophobic
12	ARG 310	VAL 1	4.211	Hydrophobic
13	TRP 7	ARG 509	4.164	Hydrophobic
14	TRP 7	ARG 427	5.343	Hydrophobic
15	TRP 7	ARG 509	4.643	Hydrophobic
16	TRP 12	LYS 336	5.125	Hydrophobic

### De novo structure prediction

**Figure 7:**
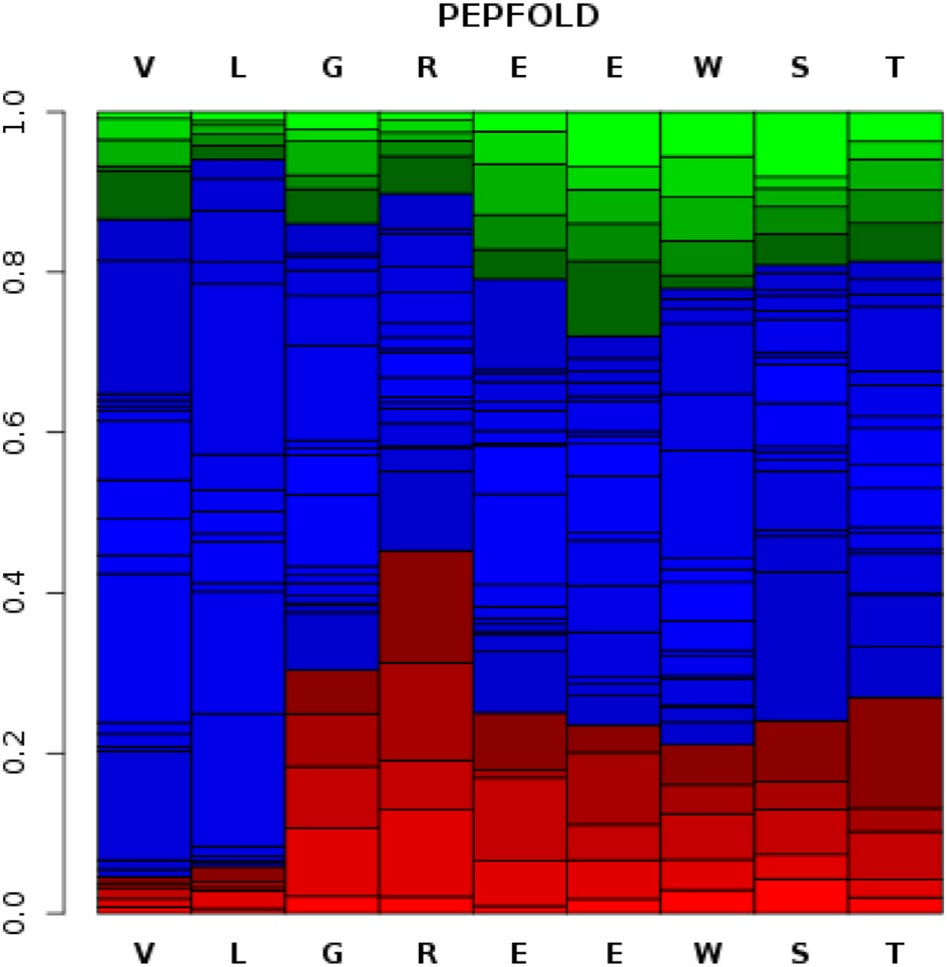
De novo conformation prediction for the VLGREEWSTSYW peptide. The de novo conformation of the VLGREEWSTSYW peptide shows variable possibilities for each amino acid residues to attain a secondary structure: α-helix (red), coil (blue), and extended conformation (green).

The de novo conformation of the VLGREEWSTSYW peptide used in the current experiment shows varying possibilities to achieve a secondary structure. The result of the de novo prediction gives an assessment of the most probable conformations in neutral medium. The sOPEP score predicts that most of the region will be coiled (Fig. 7).

The input structures initially generated for the peptide–protein docking were given all the possible conformations and subsequently resulted in a flexible loop. The simulation gave an unstructured conformation that exhibited affinity toward EGFRvIII (Fig. 6).

## Discussion

Cancer cells often display high numbers of certain cell surface molecules, such as tumor associated antigens or specific receptors, that infrequently occur in normal tissues and represent potential targets for tumor diagnosis and treatment. EGFRvIII is present in a number of human malignancies: ovarian, breast cancer and glioblastomas and has never been identified in normal tissue. Thus, the mutated receptor is an obvious choice as a target for development of new cancer-specific ligands. Therefore, the screening and identification of peptides that specifically bind to the EGFRvIII receptor will aid the development of novel probes for cancer detection and therapy. For this purpose, we used a 12-mer phage display peptide library to obtain peptides that specifically bind to the EGFRvIII receptor. Giving that the end goal is to use the EGFRvIII specific peptide in targeted drug delivery systems, the critical parameter is the specificity of the targeting on cells. Conformational epitope targeting is a key concept for specific targeting without normal tissue toxicity. In fact, EGF receptors in solution are predominantly present as a monomer in unstimulated conditions. The process of EGFR activation consists of multiple steps: ligand-induced conformational change and dimerization of EGFR lead to trans- and auto-phosphorylation of the tyrosine residues. This allows an exposure of certain epitopes called conformational epitopes. Activation of EGFR is generally caused by the binding of ligands. However, the conditions that allow for the exposure of this epitope preferentially occur under tumor-specific conditions as a result of EGFR overexpression.

The most common screening strategy involves using a purified specific target, absorbing it to a solid phase, and screening for binding by ELISA. With this method, the peptides selected using purified recombinant protein might not be capable of accessing their targets in living cells. Furthermore, sequencing usually only performed for the last round due to cost and time related factors, masking the analysis of different peptide hits and subsequent enrichment over rounds. To minimize this risk, we employed direct capture panning on the extracellular active form of the EGFRvIII, so conformational epitopes are also displayed in solution transiently. For peptide selection from the eluted phage, we used high-throughput sequencing to analyze the abundance and diversity of isolated peptides, as well as to identify target-binding peptide motifs. Therefore, we were able to select peptides that bind to this transient conformation. We further confirmed the binding using phage displayed peptides on intact and viable cells stably expressing EGFRvIII receptors as target cells, which ensures a more specific target and the isolation of a peptide that can specifically bind to cells expressing EGFRvIII receptors.

In this study, we selected five peptides from the third round of biopanning using the top hits identified by high-throughput sequencing analysis according to their enrichment over rounds. We performed peptide cloning of the selected peptides and sequencing validation prior to cellular flow cytometry. Subsequently, the five phage-displayed peptides were further tested using flow cytometry to confirm their specific binding to EGFRvIII cells. To decrease non-specific binding for each phage displayed peptide selected for binding confirmation, the amplified phages were mixed with absorber parental HEK cells before incubation with EGFR WT and EGFRvIII cells. Among these five phage-displayed peptides, VLGREEWSTSYW appeared to show specific binding to the EGFRvIII cells compared to the EGFR WT and the parental cell giving confidence that the phage was binding to the cell-surface exposed region of its respective target antigen. Therefore, this peptide was selected for further investigation. A multiple sequence alignment showed that the sequence did not exhibit homology to the sequences of any characterized proteins in various protein databases. This finding demonstrates that VLGREEWSTSYW is a novel peptide and may mimic a complex epitope, which may explain why it is not present in any database.

The docking results suggest that the identified peptide, targets the human cancer-specific EGFRvIII. The EGFRvIII extracellular domain structure was modelled by homology modelling. The secondary structures of the peptides were predicted and docked on the active sites of EGFRvIII.

In this paper, we showed that when using cells overexpressing EGFRvIII or EGFR WT, VLGREEWSTSYW peptide has a better selectivity to EGFRvIII cells than to EGFR WT cells compared to FALGEA (which is the only EGFRvIII selective peptide reported to date). The flow cytometry and MST experiments showed that VLGREEWSTSYW displays greater affinity in cell surface binding experiments despite weaker binding in solution and demonstrate that both peptides bind to EGFRvIII while they do not bind specifically to EGFR WT either in solution or on cells. We note that due to the differences between solution and cell surface binding, the Kd of peptide ligands for solution phase EGFR receptors have typically not been reported in prior literature. The fact that our MST results show a ~ 150-fold higher Kd for FALGEA to EGFRvIII (Kd 3.7 + /− 73.8 µM), compared to its reported cellular Kd of 23 nM^22^, corroborates the idea of conformational epitope differences between active receptors on cells and quiescent receptors on solution. Indeed, despite VLGREEWSTSYW displaying a higher Kd for solution phase EGFRvIII than FALGEA, the mean fluorescence observed in cellular binding was significantly higher for the former.

Interestingly, we observed by computational docking that the VLGREEWSTSYW peptide showed the highest binding affinity towards EGFRvIII compared to all the other peptides tested. The negative hit identified by phage flow cytometry also showed no significant binding in silico. This corroborated our wet lab data and further guarantees the credibility of the VLGREEWSTSYW peptide being an EGFRvIII-specific peptide.

Even though the binding to EGFRvIII is relatively weak with a single VLGREEWSTSYW peptide, the results are still very interesting for targeted drug delivery systems because the avidity of the peptide to the target will be increased by multivalence when using multiple copies of this peptide on a same nanoparticle system. In addition, we observed significantly less nonspecific cellular binding for the VLGREEWSTSYW peptide compared to the YHWYGYTPENVI peptide.

Thus, VLGREEWSTSYW warrants further investigation regarding its biological characteristics. First, we confirmed VLGREEWSTSYW specificity of the EGFRvIII cells by flow cytometry binding experiments with the free C-terminal FITC labeled peptide. While we saw a weaker signal with the free C-terminal FITC labeled peptide compared to the phage displayed format of the same peptide, this could be explained by the multimeric display of the phage particle (as in the case of nanoparticles) which is known to increase avidity and may increase the labeling signal on cells. Because VLGREEWSTSYW is a novel peptide, it will be necessary to determine whether VLGREEWSTSYW can act as an antagonist to the cancer-specific EGFRvIII receptor and understand the biological function of the peptide. Therefore, additional research examining whether the VLGREEWSTSYW peptide can act as a vector facilitating the transfer of chemotherapeutics or act as an inhibitor of the cancer-specific EGFRvIII receptor expressed in various cancers should be performed with the aim of developing VLGREEWSTSYW as a novel targeting probe for the imaging and treatment of brain tumors. This study provides a basis for the further development of peptide ligand-based human EGFRvIII receptor-targeted tumor diagnosis and treatment.

## Materials and methods

### Phage selection

Ph.D.™-12 Phage Display Peptide Library Kit was purchased from New England Biolabs Inc. (Beverly, MA, USA). Biopanning procedures were done according to the manufacturer’s instruction with certain modifications. Briefly, a 96-well plate was coated with 150 µL hEGFRvIII (Sino Biological #29662-H08B) (in the first two rounds at 100 μg/mL, the third round at 10 μg/mL) overnight at 4 °C. Wells were washed with PBS, blocked with blocking buffer (3% BSA in PBS), washed six times with cold PBST (PBS + 0.1% [v/v] Tween-20), then incubated with 10^10^ pfu phage peptide library Ph.D.™-12 for 2 h at RT. Unbound phages were removed by washing 10 times with cold PBST (PBS + 0.1% [v/v] Tween-20 in the first round and 0.5% [v/v] in the other two rounds). Bound phages were eluted with 100 μL of 0.2 M Glycine–HCl (pH 2.2), 1 mg/mL BSA and neutralized with 15 μL of 1 M Tris–HCl, pH 9.1. The elution procedure was repeated three times and the final eluate was used for amplification in Escherichia coli ER2738 culture. Recovered phages were subjected for two more rounds of biopanning with hEGFRvIII proteins (Sino Biological #29662-H08B). The eluates of each round were titrated by qPCR to enumerate phages and eluate from the third round of screening for NGS sequencing. Streptavidin was used as a biopanning control with the same condition as the target except for elution. The unbound phages were eluted with 100 μL of 0.1 M Biotin in PBS for 30 min.

### Phage titration by qPCR

We used the quantitative polymerase chain reaction (qPCR) method to enumerate M13 phage particles from biopanning. Since each individual phage particle can only contain one copy of genomic DNA (ssDNA), one phage genome is equivalent to one phage particle; by quantifying the number of phage genomes, it is feasible to quantify the number of phages and differentiate between infectious and non-infectious phages with DNase I pre-treatment of the phage samples. During qPCR, fluorescent reporter dyes bind phage genomic DNA through sequence-specific primers during PCR amplification, and the fluorescence signal (relative fluorescence unit, RFU) increases with each round of amplification. When the fluorescence signal reaches the threshold, that round/cycle of amplification is noted as the threshold cycle (Ct) (Fig. S1A). The known concentrations of reference phage DNA were plotted against their Ct values to establish a standard curve (Fig. S1B,C).

The following Eq. (1) was used to convert the M13 phage reference DNA concentrations from fg/μL to genome copies (gc) per unit of volume (gc/µL):

1 $$M13\, phage \, DNA \, concentration\, in\, \frac{gc}{\upmu L}=2*\left(\frac{\left[ssDNA\, (\frac{\rm g}{\upmu L})\right]}{\left[DNA \, size\, \left(bp\right)*607.4\, +\, 157.9\right)}\right)*(6.02*18)$$

Note: bp: base pair; reference M13 genomic DNA is 7222 bp.

The concentration of M13 phages in the biopanning samples (target or background) were calculated using the linear regression Eq. (2) from the standard curve (R^2^ = 0.999):

2 $$Y = -3.518X + 38.069$$

Forward and reverse primers were designed upstream of the library variable region to amplify M13 phage genomic DNA (Table S1).

New dilutions for plasmid standard curves were prepared from stocks for each assay as following: M13KE plasmid with known concentration to 10 ng/µL (10^7^ fg/µL) was diluted ten-fold serial dilution for seven times until reaching 1 fg/µL. 20 μL of each M13 clone (phage) selected from bio-panning against the target were mixed with 1.25 units of DNase I (0.5 μL) and incubated at 37 °C for 10 min and then incubate at 100 °C for 15 min to stop the reaction. A background well without target was used as a counter screening. The samples were let to cool down slowly at room temperature (RT) before the qPCR reaction.

All qPCRs were performed in 10 µL reactions (8 μL PowerUp™ SYBR™ Green (Bio-Rad), 0.5 µL of 10 μM primer sets and 2 µL template. Standards and unknown samples were assayed in triplicate using the thermocycler cycling conditions set up on the C1000 Touch™ thermal cycler (^®^BioRad) as follows for a sample volume of 10 μL: one cycle at 50 °C for 2 min, one cycle at 95 °C for 2 min, followed by 40 cycles of (95 °C for 15 s, 60 °C for 1 min). After, a melt curve was running with following settings: one cycle of 95 °C for 15 s, 60 °C for 1 min, and 95 °C for 15 s.

### NGS library preparation

The library preparation is done in compliance to Illumina NGS library generation protocol. The elution samples from each round of biopanning were subjected to two PCRs. The first PCR adds the barcodes necessary to demultiplex the DNA sequences during data analysis and the second PCR adds the adapter and reading sequences that are important for sequence reading by the iseq100 (Fig. S6). Phage sample, 20 μL, was added with 0.5 μL (1.25 units) DNaseI and heated at 37 °C for 10 min to degrade all residual DNA in the mix. Following which, the reaction was heated at 100 °C to stop the DNAse activity and degrade the capsid to release the ssDNA. This ssDNA serves as the template for the first PCR. ssDNA, 10 μL was mixed with 2.5 μL (5 pM) forward and reverse primers, 0.5 mM dNTPs, 10 μL GC enhancer buffer, 10 μL polymerase buffer and 1U of Platinum SuperFi DNA polymerase (Thermofisher Scientific). The PCR cycle was set to one cycle at 98 °C for 30 s followed by 25 cycles at 98 °C for 15 s, 60 °C for 1 min and 72 °C for 1 min. The PCR products were viewed on a 1% agarose gel and any other contaminant bands were discarded by gel purification on a column using a NEB PCR clean up kit. The separate PCR products were pooled and used as a template for the second PCR. The conditions for the 2nd PCR were like the 1st PCR except that only 15 cycles were run. Following which, the PCR products (expected size 200 bp) were analyzed on a 1% agarose gel and purified. The concentration of DNA was determined using a dsDNA High Sensitivity Assay Kit (Thermofisher Scientific) following the manufacturer’s protocol. According to Illumina recommendation, 50 pM of DNA spiked with 10% PhiX control was fed into the flow cell for sequencing by iseq100. The primers used in PCR1 are NGS-1, NGS-2, NGS-3, NGS-4 and NGS Rev. The primers used in PCR2 are seq fwd. and seq rev (primer sequences are provided in Table S2).

### NGS data analysis

The NGS data analysis is an imperative approach to filter out all the non-specific binders and identify the putative binders. NGS in combination with phage display is used widely these days as it gives us a global idea of how thousands of peptide sequences evolve in subsequent rounds of biopanning unlike Sanger sequencing which only yields sequences of a few clones. This is highly useful as we can follow how the read of a peptide evolves with the rounds of biopanning. The iseq100 generates a fastq file which provides us with the sequence and phred score. There are several programs which are executed sequentially to follow each step of the analysis that is shown in Fig. S7. The overall quality of the NGS sequencing data is first checked using the FASTQC software. The amplicon of interest is a 200 bp including the barcode sequence and the adapter sequence and contains an Eag1 and Kpn1 site. The DNA sequence corresponding to the peptide insert is located in between these restriction sites. The first program extracts only those DNA sequences that contains these restriction sites and has a phred score above 30. The total number of sequences and the corresponding number of reads for each DNA sequence are recorded in a separate CSV file. The second program demultiplexes the DNA sequences into separate CSV files corresponding to their barcodes that are added in the 1st PCR amplification. Each barcode file corresponds to a specific round of biopanning. The 3rd program is executed on each barcode file where each DNA sequence is translated into the peptide sequence. Any DNA sequence without the peptide insert is discarded. Due to codon degeneracy, different codons can translate into same amino acids, thus after translation identical sequences exist with different reads. A 4th program collates all the identical sequences and their reads into one, thus refining the data. Once the final list of peptides is obtained from each barcode, the frequency of all the peptides are calculated by normalizing the reads. The frequency is the ratio of individual reads to the total number of reads in that specific round. This way, we normalize the difference in the number of reads in the different rounds of biopanning including the background. Then, the background peptides are filtered out from the peptides in all the rounds. The peptides in the last round of biopanning were sorted according to their enrichment values. The enrichment is the ratio of the frequency of the peptide in the last round to the first round of biopanning. The top 20–30 peptide hits that evolved over the rounds are chosen for further characterization. All the frequency and enrichment calculations are done by command lines written in MS Excel.

### Cell lines and cell culture

Cell cultures, flow cytometry experiments with phages or peptides, and spectrofluorometer experiments were performed by the flow cytometry core facility at Institut Mondor de Recherche Biomédical (IMRB U955, France). 293T human embryo kidney cells (Sigma; #85120602-1VL), CLTH/EGFR WT cells (Celther; #CL 01010-CLTH) and CLTH/EGFRvIII cells (Celther; #CL 01001-CLTH) were purchased from Sigma and Celther accordingly. CLTH/EGFR WT and CLTH/EGFRvIII cell lines are established from human embryo kidney cells to stably express EGFR WT and EGFRvIII proteins respectively. The three types of cells were maintained in Dulbecco’s modified Eagle media (high glucose) supplemented with 10% fetal bovine serum (FBS), 100 U/mL penicillin, 100 mg/mL streptomycin and 1% MEM NEAA. The cells were cultured at 37 °C in a humidified atmosphere containing 5% CO_2_.

### Flow cytometry

Prior to being tested with the selected phages or peptides, each cell line was incubated with different commercial antibodies to confirm their phenotype (Fig. S3). A monoclonal anti-EGFR WT antibody (Life Technologies) and an anti-EGFRvIII primary antibody (Millipore) were used in flow cytometry experiments to evaluate the expression of EGFR WT and EGFRvIII receptors on CLTH/EGFR WT cells and CLTH/EGFRvIII cells respectively. Anti-IgG1 conjugated to phycoerythrin (PE) (Miltenyi Biotec) was used as a secondary antibody to read the fluorescence of PE. 293T cells were tested as a negative control. A phage-free control experiment was also performed on each cell type prior to testing phages. Anti-M13 conjugated to biotin (Abcam) was used as a primary antibody, anti-mouse IgG3 kappa monoclonal conjugated to biotin (Abcam) as isotype control and anti-biotin conjugated to PE (Miltenyi Biotec) as secondary antibody. A list of the antibodies is available in Table S3.

### On phages

Binding of the selected phages on 293T human embryo kidney, CLTH/EGFR WT and CLTH/EGFRvIII cells was characterized by flow cytometry. Approximately 1 × 10^6^ 293T cells (expressing endogenous levels of EGFR WT) were collected, washed with cold PBS and blocked with 2%w/v milk-PBS (MPBS), rotating for 30 min at 4 °C. Concurrently, approximately 10^10^ phages from the rescued library stock were also blocked in MPBS. The blocked phages were then added to the blocked cells and incubated rotating for 1 h at 4 °C. After centrifugation at 500*g* for 3 min, the supernatant containing non-bound phages was collected. This step allowed the removal of phages that interacted with this EGFR negative cell line (i.e. not an EGFR-specific binding). The supernatant was then used to resuspend transfected cells washed beforehand (CLTH/EGFR WT expressing EGFR WT and CLTH/EGFRvIII expressing EGFRvIII). Treated cells were incubated for 1 h at 4 °C, collected by centrifugation and washed two times with PBS at pH 7.4. Cells were stained with Viobility™ 405/520 fixable dye (Miltenyi Biotec) as a viability marker. Cells were analyzed by flow cytometry on a MACSQuant Analyser 16 (Miltenyi Biotec) using the MACS Quantify software to acquire and process the data. Viobility™ 405/520 fixable dye was excited at 405 nm and fluorescence was collected with the 525/50 nm filter. PE was excited at 488 nm and fluorescence was collected with the 579/34 nm filter. Viable cells were gated, and PE fluorescence intensity was recorded.

### On FITC-Ahx-peptides

Flow cytometry analysis was also performed to quantitatively analyze the binding affinity of the synthetized peptides to the different cell types. Briefly, 293T, CLTH/EGFR WT and CLTH/EGFRvIII cells (100,000 cells, 500 µL) were plated in 24-well plates and incubated overnight at 37 °C in a humidified atmosphere containing 5% CO_2_ overnight. The next day, cells were treated with FITC-Ahx-peptides using a stock solution in pure DMSO at 5 mM to reach a final concentration of 1, 5 or 10 µM (final percentage of DMSO of 0.2%). Plates were incubated for 20 min at RT. Cells were detached from the plates by a PBS wash, transferred to FACS tubes and washed two times with 4 mL of PBS. Cells were stained with LIVE/DEAD™ Fixable Near-IR Dead Cell Stain Kit (Thermofisher) as a viability marker. Cells were analyzed by flow cytometry on a MACSQuant Analyser 16 (Miltenyi Biotec) using the MACS Quantify software to acquire and process the data. LIVE/DEAD™ Fixable dye was excited at 640 nm and fluorescence was collected with the 785/62 nm filter. FITC was excited at 488 nm and fluorescence was collected with the 525/50 nm filter. Viable cells were gated, FITC fluorescence intensity was recorded and the mean fluorescence intensity (MFI) was measured. MFI values were then normalized by subtracting MFI from blank cells and dividing by the correction factor determined using a spectrofluorometer.

### Peptide and FITC-Ahx-peptide synthesis

The candidate peptides were synthesized by ProteoGenix (France) using standard solid-phase Fmoc chemistry. FITC was conjugated to the N-terminus of each candidate peptide with a 6-aminohexanoic acid (Ahx) linker. The products were purified to a minimum purity of 95% by high-performance liquid chromatography (HPLC) and isolated by lyophilization. The sequence and structure of each peptide were characterized by mass spectrometry (MS) and nuclear magnetic resonance spectroscopy (NMR), and the purity of the peptides was determined by analytical HPLC. Results are shown in Figs. S8, S9 and S10 and Table S4.

### Spectrofluorometer on FITC-Ahx-peptides

Since FITC fluorescence can be impacted by the nature of the amino acids in the peptide sequence and since the conjugation rate of FITC in the peptide synthesis can fluctuate, it was needed to measure a correction factor according to the fluorescence intensity of each peptide. As flow cytometry cannot analyze fluorescence of small molecules, a spectrofluorometer was used in similar conditions (excitation and emission) to the flow cytometry conditions. Experiments were performed by the flow cytometry core facility at Institut Mondor de Recherche Biomédical (IMRB U955, France). Fluorescence intensities at 525 nm of the FITC-Ahx-peptides in PBS were measured using a Varioskan plate reader (Thermo Scientific) and the SkanIt Software 5.0. Samples with concentrations between at 0.625, 1.25, 2.5, 5, 10 and 20 µM were prepared in triplicates using stock solutions of peptides in DMSO at 5 mM. Samples (100 µL) were analyzed in a 96-well plate using the following conditions: temperature of 25 °C, excitation wavelength at 490 nm, excitation bandwidth of 5 nm, emission wavelength at 525 nm, by step of 1 nm, measurement time 100 ms, and upper lens. Linear regressions were determined for each peptide. The correction factor was determined by dividing each slope by the arbitrary value 100 and was used to normalize the MFI obtained by flow cytometry. Results are shown in Fig. S11 and Table S5.

### Computational protein-peptide docking study

The computational docking study between the protein and the peptide is a complex and rigorous method. The wild type EGFR protein was downloaded from RCSB-Protein Data Bank with ID: 3NJP (Extracellular and Transmembrane Domain Interfaces in Epidermal Growth Factor Receptor as initial structure)^32^. The protein was further prepared for computational usage in Chimera software without disturbing the back bone of the protein^33^. The protein is a dimer and has two chains, therefore truncation was executed on both the chains. The region from VCQGT to CVKKCPR was sliced and saved as separate PDB file. The N-terminal LEEKKG part was retained by building the amino acids. The truncated protein dimer was relaxed by energy minimization using Swiss PDB viewer by applying GROMOS forcefield^34,35^. The conformation with local minima was considered for peptide docking in further docking studies. The active site of the protein in dimer form of EGFRvIII was predicted using CastP server tool^36^. The peptides were built as linear fragments to give high flexibility during docking. Using HPEPDOCK server^37^, global docking was performed and 100 conformations per each peptide were generated. Similar methodology will also be followed to the peptide that shows affinity towards EGFRvIII and EGFR-WT for cross verification of peptide specificity. The top scored ligands as well as the ligands that show affinity towards the identified active sites were sorted. The number of conformations of peptide that shows affinity to a protein is a parameter for peptide specificity. The conformation that exists at the active site were ranked based on score value. The model lowest binding affinity value was considered as best conformation for further analysis and interaction data with the target protein.

### De novo structure prediction

PEP-FOLD executes the de novo prediction of peptide conformation from an amino acid sequence, based on the Hidden Markov Model. The PEP-FOLD 3D generation is performed using a discrete set of structural prototypes, and using the sOPEP coarse-grained force field^38,39^. The amino acid sequence was subjected to approximately 100 simulations in PEP-FOLD. Each simulation samples various regions of conformational space that give the probability of appearance of helix or other structural features along the sequence. This result is for highly probable conformations that could occur in a neutral pH medium.

### Microscale thermophoresis

Microscale thermophoresis (MST) were performed on a Monolith NT.115 Pico (red-pico) at 25 °C, with 5% LED power and 40% laser power. Proteins were labelled with NT 650 NHS 2nd Gen (NHS chemistry), 10 nM (final concentration) of protein was used in the experiments. A serial of 1:1 dilution with 16 concentrations of peptide was prepared. 5 μL of each dilution step were mixed with 5 μL of the fluorescent molecule. The mixture was filled in premium coated capillaries. The highest concentration (final concentration) of VLGREEWSTSYW peptide used in the experiments was 50 µM. The highest concentration (final concentration) of FALGEA peptide used for EGFR WT and EGFRvIII experiment was 20 µM and 10 µM respectively. The experiments were performed in an assay buffer containing 1× PBS pH 7.4, 0.005% Tween-20, 1% DMSO. Three independent experiments were performed for each condition.

## Supplementary Information

Supplementary Information.
