# Supplementary material for: Identification of a novel peptide ligand for the cancer-specific receptor mutation EGFRvIII using high-throughput sequencing of phage-selected peptides
Source: Sci Rep. 2022 Dec 1;12:20725. doi: 10.1038/s41598-022-25257-4 (PMC9715707; doi:10.1038/s41598-022-25257-4)
Supplement: Supplementary file 1 — Supplementary Information. [file 41598_2022_25257_MOESM1_ESM.docx]

**SUPPORTING INFORMATION**

**Identification of a Novel Peptide Ligand for the Cancer-Specific Receptor Mutation EGFRvIII Using High-throughput Sequencing of Phage-selected Peptides**

**S Mansour^a^, I Adhya^a^, C Lebleu^a^, R Dumpati^b^, A Rehan^a^, S Chall^b^, J Dai^a^, G Errasti^a^, T Delacroix^a^ and R Chakrabarti ^a,b,c^***

^a^ Center for Protein Engineering & Drug Discovery, PMC Isochem SAS | 32, rue Lavoisier F-91710 |Vert-Le-Petit| France

^b^ Division of Computational Research, Chakrabarti Advanced Technology, Hyderabad, Telangana, India

^c^ Chakrabarti Advanced Technology, LLC **|** PMC Group Building, 1288 Route 73, Ste 110 **|** Mount Laurel, NJ 08054|USA

*To whom correspondence should be addressed: [raj@pmc-group.com](mailto:raj@pmc-group.com)

ORCID:

Sourour Mansour 0000-0003-1959-1341

Coralie Lebleu 0000-0003-1843-8599

Santu Chall 0000-0003-3245-6902

Jingqi Dai 0000-0001-6294-8524

Gauthier Errasti 0000-0001-7571-3257

Thomas Delacroix 0000-0001-8932-6899


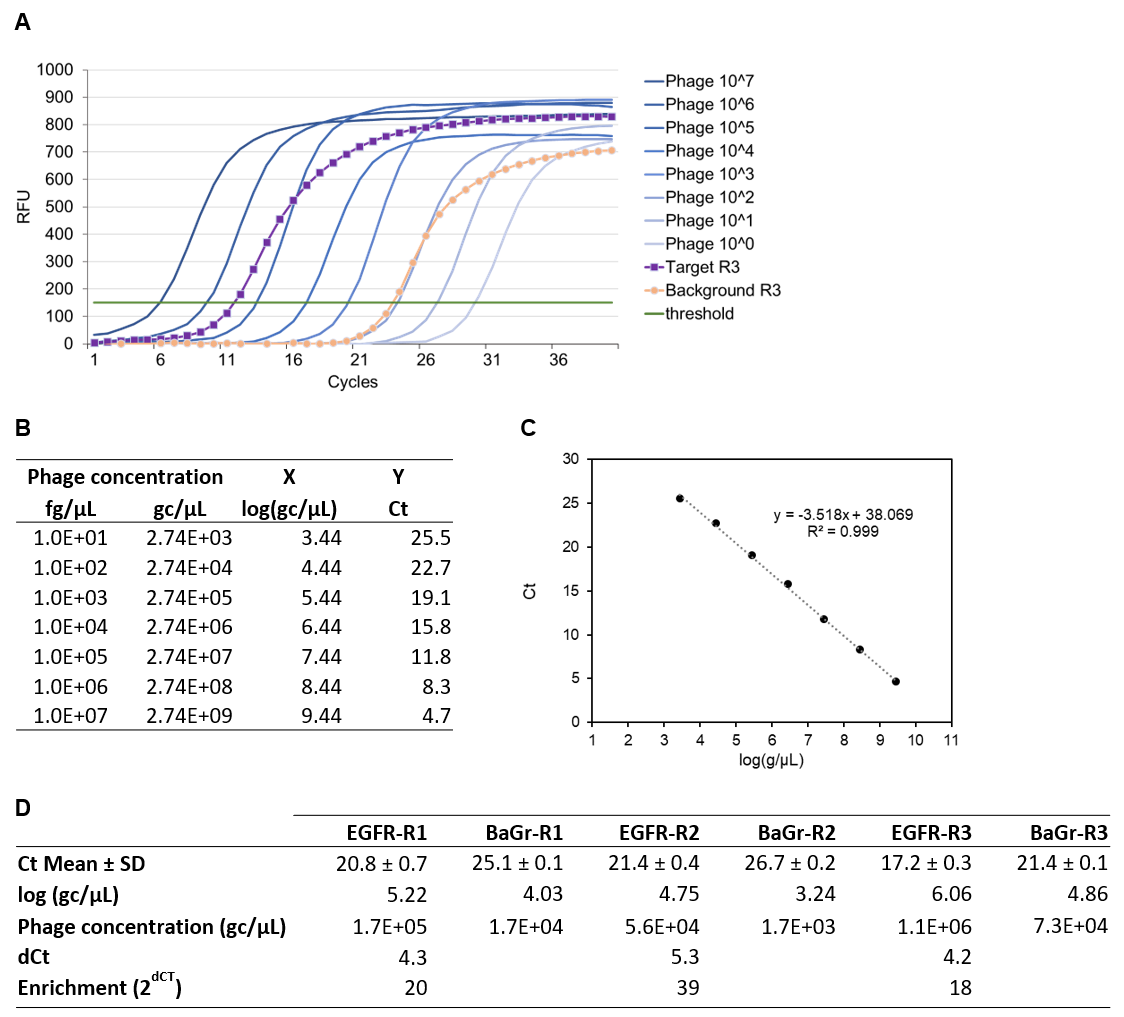


**Figure S1. Analysis of raw data of qPCR to determine the phage concentration and enrichment.** A) Relative fluorescence units (RFU) versus cycles for standard samples (blue) and round 3 target (purple) and background (orange) to determine threshold cycle (Ct) values. Threshold is indicated in green. B-C) Ct values for the standard samples were plotted against the logarithm transformation of the known DNA concentrations in gc/μL to get a standard curve. D) The standard curve was used to calculate the phage concentration in gc/μL of the unknown concentrations of phage samples based on their Ct values. Difference of Ct values between target and background (dCt) was used to calculate the enrichment (2dCT).


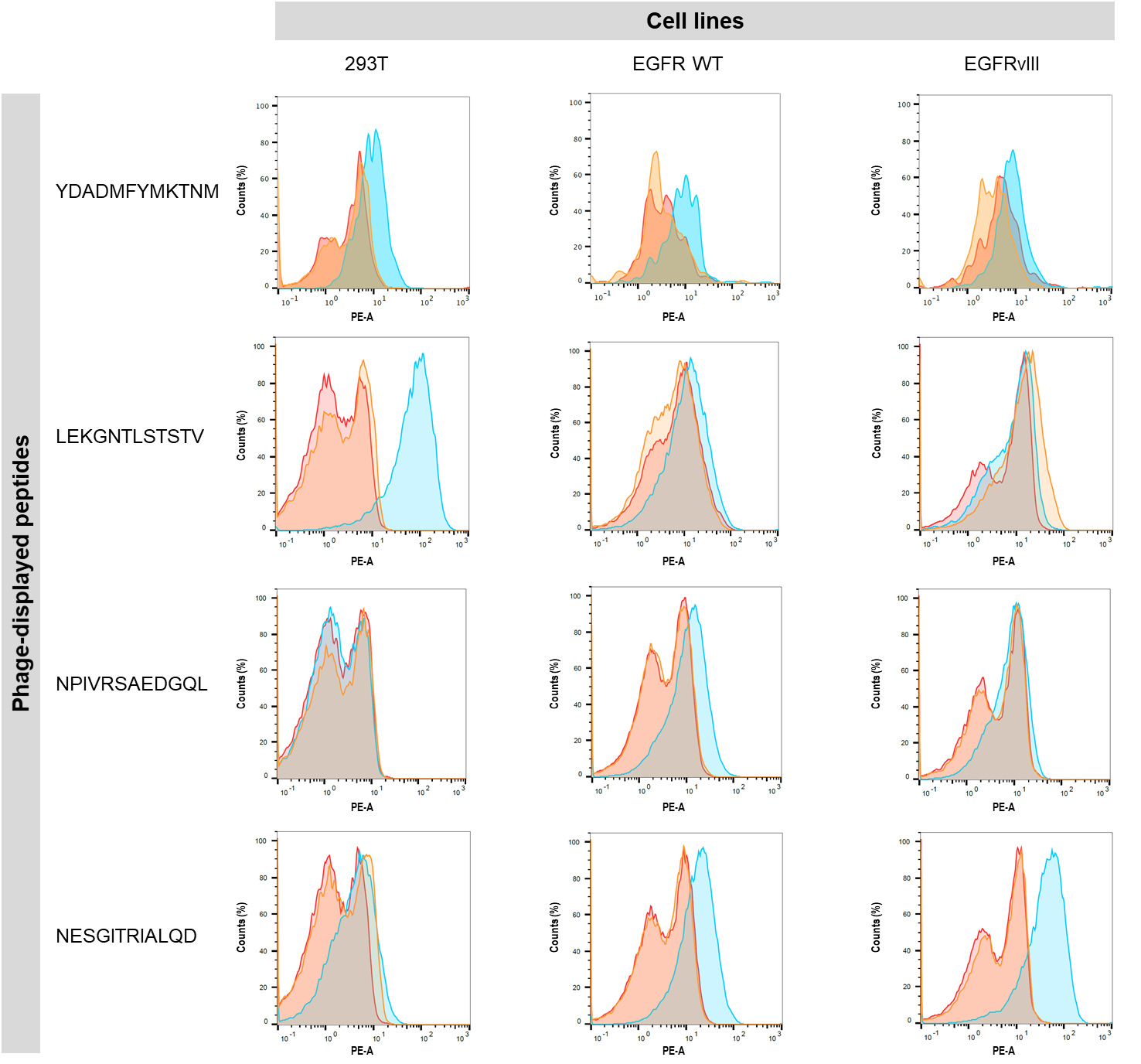


**Figure S2. Binding analysis of phage-displayed peptides by flow cytometry**. 293T, EGFR WT and EGFRvIII cell lines were incubated with the different phage-displayed peptides. Cell counts in % versus PE signal: when incubated with primary + secondary antibody (anti-M13_biotin + anti-biotin_PE, orange), isotype labelling + secondary antibody (anti-IgG3_biotin + anti-biotin_PE, blue) or secondary antibody only (anti-biotin_PE, red).


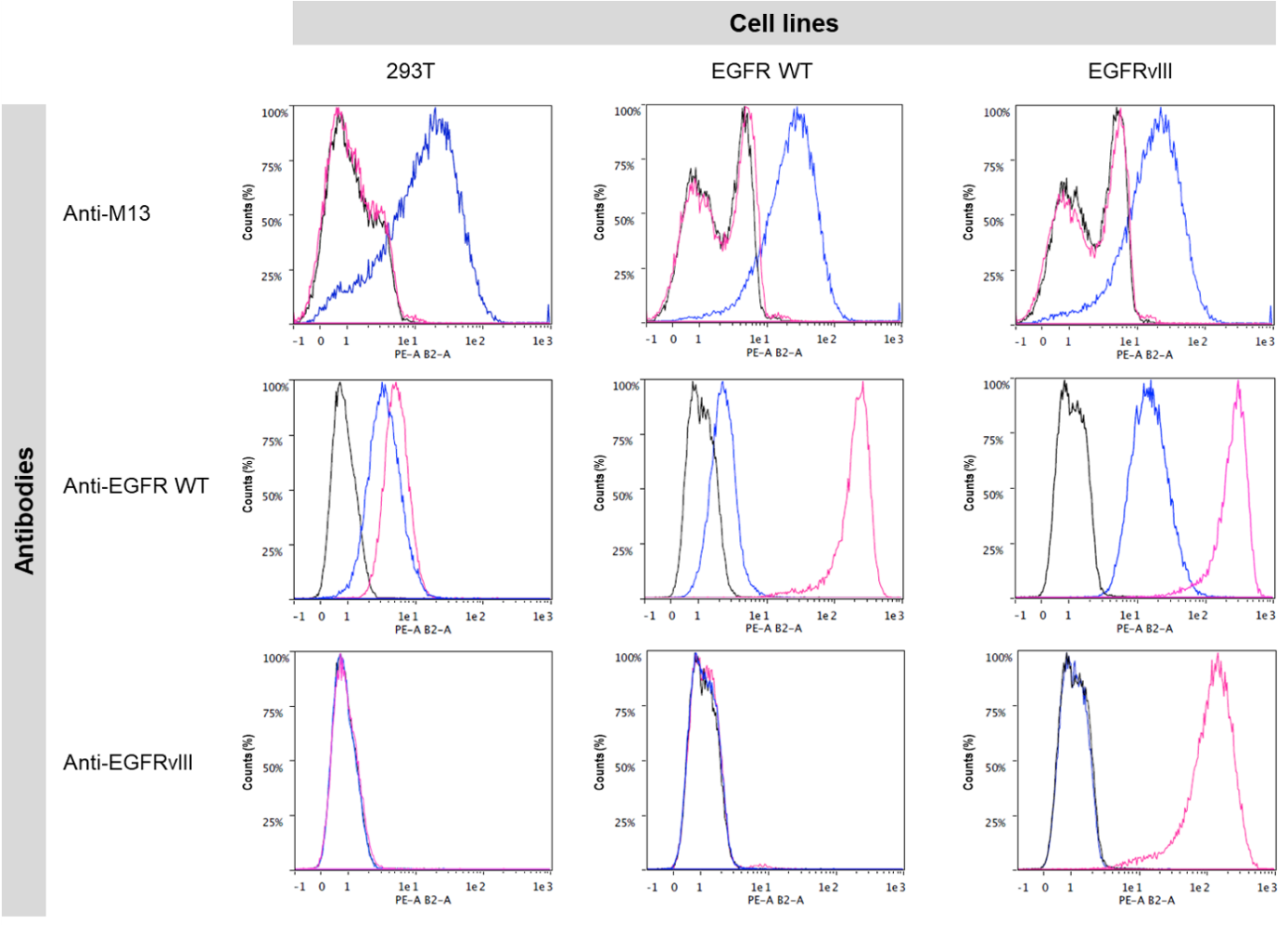


**Figure S3. Fluorescence intensity quantification measured by flow cytometry.** 293T, EGFR WT and EGFRvIII cell lines were incubated either with anti-M13, anti-EGFR WT or anti-EGFRvIII as primary antibodies. Cell counts in % versus PE signal: when incubated with primary + secondary antibody (pink), isotype labelling + secondary antibody (blue) or secondary antibody only (black).

**
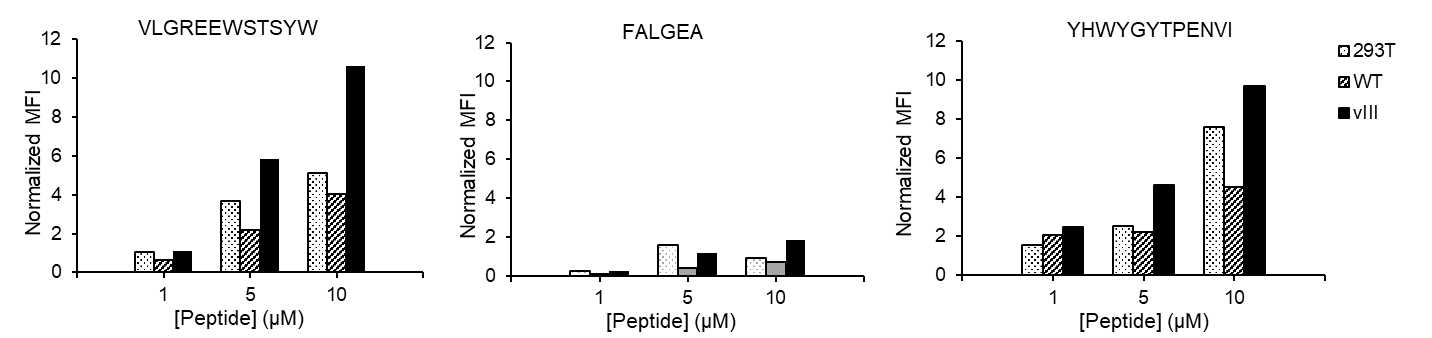
**

**Figure S4. Quantitative cellular binding of FITC-Ahx-peptides with different concentration on 293T, WT and vIII cells by flow cytometry.** Normalized mean fluorescence intensities (MFI) of each peptide for each cell line are presented for 3 different concentrations of peptide (1, 5 and 10 µM).

*****
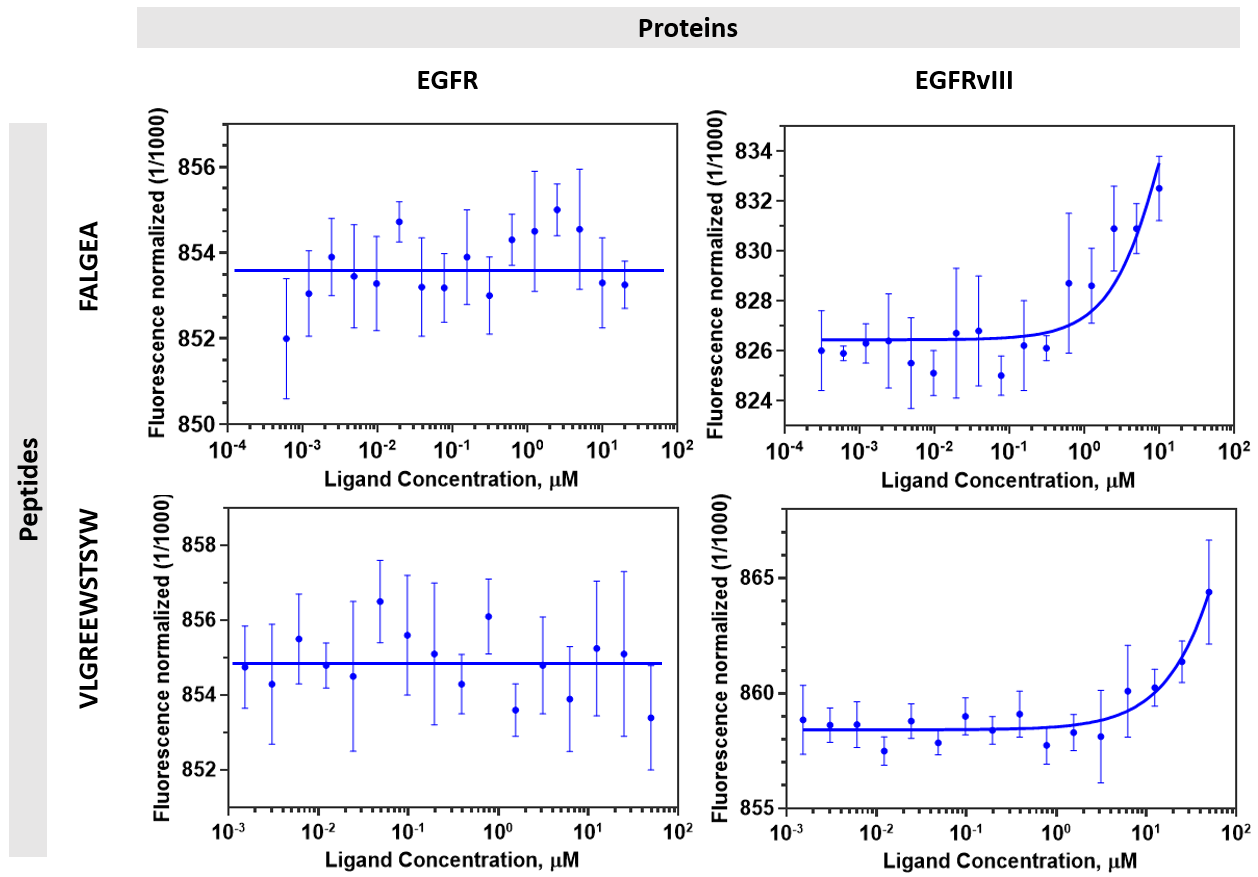


**Figure S5. Analyses of interactions of EGFR WT (left panel) and EGFRvIII (right panel) in solution phase with the peptides FALGEA (top row) and VLG (bottom row) by MST.** MST were performed on Monolith NT.115 Pico (red-pico) at 25 °C, with 5 % LED power and 40 % laser power. Proteins were labelled with NT 650 NHS 2nd Gen (NHS chemistry), 10 nM (final concentration) of protein was used in the experiments. A serial of 1:1 dilution with 16 concentrations of peptide was prepared. 5 μL of each dilution step were mixed with 5 μL of the fluorescent molecule. The mixture was filled in premium coated capillaries. The most concentrated (final concentration) VLGREEWSTSYW peptide used in the experiments was at 50 µM. The highest concentration (final concentration) of FALGEA peptide used for EGFR WT and EGFRvIII experiment was 20 µM and 10 µM respectively. The experiments were performed in an assay buffer containing 1x PBS pH 7.4, 0.005% Tween-20, 1% DMSO. Three independent experiments were performed for each condition. Higher concentrations were not tested due to solubility limits. The estimated Kd’s for EGFRvIII were: Kd,FALGEA = 3.7 ± 73.8 µM ; Kd,VLGREEWSTSYW = 361.5 ± 1172.0 µM.

**Table S1. Primers used for qPCR.**

| **Primer name** | **Primer sequence** |
| --- | --- |
| QPCR-F | ATG CAA GGT ACC TTT CTA TTC TCA CTC T |
| QPCR-R | GTA TGG GAT TTT GCT AAA CAA C |


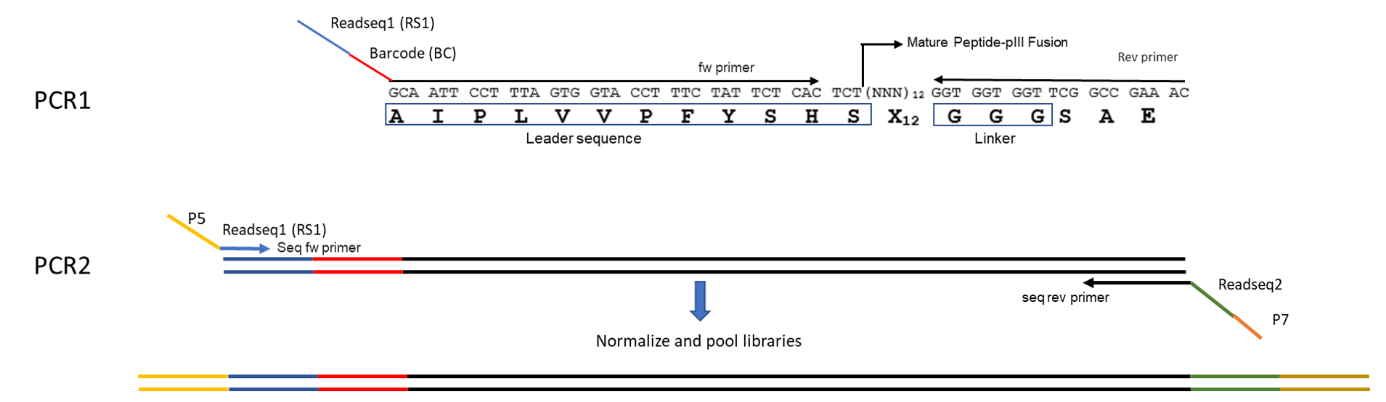


**Figure S6. Primer design for Illumina sequencing of peptide libraries.** For PCR 1: forward and reverse primers that are complementary upstream and downstream of the region flunking the peptide are designed with overhang adapters and used to amplify templates from phage DNA. For PCR2: A subsequent limited cycle amplification step is performed to add multiplexing indices and Illumina sequencing adapters. Libraries are normalized and pooled and sequenced on the iSeq100 system.

**Table S2. Primers used for PCR1 and PCR2 in NGS library preparation.** The barcodes are shown in red. The barcodes enable demultiplexing during NGS data analysis.

| **Primer name** | **Primer sequence** |
| --- | --- |
| NGS - 1 | CTCTTCCGATCTATGCCTTACGGATCCTCATTAATGCAAGCAATTCCTTTAGTGGTACCTTTCTATTCTCAC |
| NGS - 2 | CTCTTCCGATCTATGCCTTACGGATCCTCATTAATAAGCGCAATTCCTTTAGTGGTACCTTTCTATTCTCAC |
| NGS - 3 | CTCTTCCGATCTATGCCTTACGGATCCTCATTAGCATAAGCAATTCCTTTAGTGGTACCTTTCTATTCTCAC |
| NGS - 4 | CTCTTCCGATCTATGCCTTACGGATCCTCATTATATAGCGCAATTCCTTTAGTGGTACCTTTCTATTCTCAC |
| NGS rev | GTTTCGGCCGAACCACCACC |
| seq fw | AATGATACGGCGACCACCGAGATCTACACGCTTGTCAACACTCTTTCCCTACACGACGCTCTTCCGATCTATGCCTTACGGATCCTCATTA |
| seq rev | CAAGCAGAAGACGGCATACGAGATATCACGACGTGACTGGAGTTCAGACGTGTGCTCTTCCGATCTGTTTCGGCCGAACCACCACC |


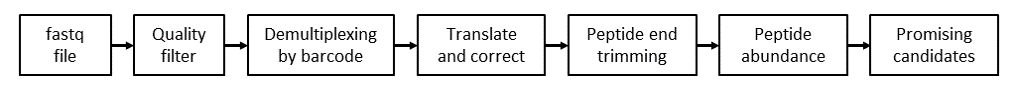


**Figure S7. Procedure for the analysis of sequencing data applying python scripts.** First, reads are separated into several files according to their barcode. Second, low-quality sequences are removed from the dataset, and remaining sequences are translated and sorted by abundance.

**Table S3. List of antibodies and controls used on 293T, CLTH/EGFR WT and CLTH/EGFRvIII cells.**

|  | **Anti-M13** | **Anti-hEGFR WT** | **Anti-hEGFRvIII** |
| --- | --- | --- | --- |
| **Primary antibody** | anti-M13 conjugated to biotin (Abcam #ab17269) | anti-EGFR WT (Life Technologies #MA5-13070) | anti-hEGFRvIII (Millipore # MABS1915) |
| **Isotype control** | mouse IgG3 kappa monoclonal conjugated to biotin (Abcam #ab18436) | mouse IgG1 kappa (Miltenyi Biotec #130-106-545) | mouse IgG1 kappa (Miltenyi Biotec #130-106-545) |
| **Secondary antibody** | anti-biotin conjugated to PE (Miltenyi Biotec #130-110-951) | anti-mouse conjugated to PE (Miltenyi Biotec #130-117-098) | anti-mouse conjugated to PE (Miltenyi Biotec#130-117-098) |

**A) FALGEA**

**B) FITC-Ahx-FALGEA**

**C) YHWYGYTPENVI**

**D) FITC-Ahx-YHWYGYTPENVI**

**E) VLGREEWSTSYW**

**F) FITC-Ahx-VLGREEWSTSYW**

**Figure S8. MS spectra of the peptides: A) FALGEA, B) FITC-Ahx-FALGEA, C) YHWYGYTPENVI, D) FITC-Ahx-YHWYGYTPENVI, E) VLGREEWSTSYW, and F) FITC-Ahx-VLGREEWSTSYW**

**A)** **FALGEA**

^1^H

^13^C

**B)** **FITC-Ahx-FALGEA**

^1^H

^13^C

**C)** **YHWYGYTPENVI**

^1^H NMR (400 MHz, DMSO-*d_6_*) δ 14.19 (d, J = 28.1 Hz, 2H), 10.77 (s, 1H), 9.17 (s, 3H), 8.95 (s, 1H), 8.78 (d, J = 7.9 Hz, 1H), 8.35 – 8.20 (m, 3H), 8.10 (dd, J = 22.6, 7.5 Hz, 2H), 8.03 – 7.97 (m, 3H), 7.93 (d, J = 8.0 Hz, 1H), 7.80 – 7.52 (m, 3H), 7.43 (s, 1H), 7.34 – 7.19 (m, 3H), 7.18 – 7.07 (m, 1H), 6.99 (dp, J = 14.5, 7.4 Hz, 10H), 6.73 – 6.56 (m, 6H), 4.66 (d, J = 7.2 Hz, 1H), 4.63 – 4.52 (m, 3H), 4.47 (t, J = 7.2 Hz, 1H), 4.47 – 4.33 (m, 2H), 4.28 – 4.02 (m, 3H), 3.97 (s, 2H), 3.87 (t, J = 6.7 Hz, 1H), 3.67 (tdd, J = 31.0, 15.8, 6.8 Hz, 3H), 3.11 (s, 0H), 2.98 (s, 3H), 3.05 – 2.85 (m, 2H), 2.78 – 2.50 (m, 4H), 2.44 (dd, J = 15.4, 6.8 Hz, 1H), 2.26 (ddd, J = 22.0, 13.9, 8.1 Hz, 2H), 2.03 (h, J = 7.2, 6.6 Hz, 2H), 1.88 (s, 3H), 1.74 (dq, J = 15.6, 7.9, 7.2 Hz, 3H), 1.43 (s, 1H), 1.19 – 0.96 (m, 4H), 0.88 – 0.76 (m, 12H).

^13^C NMR (101 MHz, DMSO*-d_6_*) δ 174.49, 173.36, 172.21, 172.04, 171.67, 171.49, 171.35, 171.30 (d, J = 7.4 Hz), 170.90, 169.37, 158.81, 158.46, 157.05, 156.26, 136.49, 134.22, 130.93, 130.70, 130.59, 128.09, 127.95, 127.77, 125.16, 124.05, 121.34, 118.75, 115.83, 115.33, 115.24, 111.70, 110.12, 67.26, 60.23 – 59.01 (m), 58.36, 57.42, 54.09, 52.41, 50.16, 37.20, 36.74, 30.67, 30.50, 29.54, 27.96 (d, J = 2.1 Hz), 24.78, 19.89, 19.61, 18.17, 15.97, 11.52.

**D)** **FITC-Ahx-YHWYGYTPENVI**

^1^H NMR (400 MHz, DMSO-*d_6_*) δ 14.04 (s, 1H), 13.93 (s, 1H), 10.74 (s, 1H), 10.13 (s, 2H), 9.99 (s, 1H), 9.14 (s, 3H), 8.93 (s, 1H), 8.31 – 8.15 (m, 5H), 8.09 (dd, J = 23.8, 7.6 Hz, 4H), 7.93 (dd, J = 20.0, 7.9 Hz, 2H), 7.73 (d, J = 8.8 Hz, 2H), 7.66 (d, J = 8.9 Hz, 1H), 7.54 (d, J = 7.9 Hz, 1H), 7.42 (s, 1H), 7.23 (dd, J = 48.4, 8.2 Hz, 2H), 7.23 (d, J = 12.0 Hz, 2H), 7.14 – 6.99 (m, 5H), 6.99 – 6.91 (m, 5H), 6.70 – 6.52 (m, 12H), 4.58 (p, J = 7.0 Hz, 4H), 4.52 – 4.28 (m, 4H), 4.28 – 3.81 (m, 4H), 3.80 – 3.25 (m, 4H), 3.07 (dd, J = 27.0, 11.2 Hz, 1H), 2.92 (dd, J = 24.3, 9.4 Hz, 1H), 2.81 – 2.64 (m, 2H), 2.62 – 2.14 (m, 18H), 2.41 (d, J = 6.8 Hz, 0H), 2.32 – 2.20 (m, 1H), 2.09 – 1.96 (m, 4H), 2.10 – 1.96 (m, 0H), 1.87 (s, 3H), 1.80 – 1.64 (m, 3H), 1.50 (d, J = 15.1 Hz, 1H), 1.60 – 1.33 (m, 4H), 1.47 – 1.35 (m, 0H), 1.28 – 1.13 (m, 2H), 1.10 (d, J = 6.3 Hz, 3H), 0.89 -0.70 (m, 11H).

^13^C NMR (101 MHz, DMSO*-d_6_*) δ 174.49, 173.36, 172.94, 172.21, 171.66, 171.49, 171.27, 170.91, 170.05, 169.38, 168.83, 159.96, 156.19, 152.35, 136.64, 133.89, 130.70, 130.58, 130.47, 129.46, 128.48, 127.79, 115.34, 115.25, 113.05, 110.20, 102.71, 57.41, 54.80, 36.73, 30.67, 28.57, 26.46, 25.45, 24.78, 19.89, 19.61, 18.17, 15.97, 11.51.

**E)** **VLGREEWSTSYW**

^1^H NMR (400 MHz, DMSO-*d_6_*) δ 11.99 (s, 0H), 10.90 – 10.58 (m, 2H), 9.14 (s, 1H), 8.59 – 7.67 (m, 11H), 7.59 (t, J = 8.3 Hz, 3H), 7.32 (t, J = 7.5 Hz, 2H), 7.23 – 6.88 (m, 11H), 6.58 (d, J = 8.1 Hz, 2H), 5.18 (s, 1H), 5.02 (s, 2H), 4.62 (q, J = 7.4 Hz, 1H), 4.49 – 4.17 (m, 10H), 4.06 (s, 1H), 3.82 (dd, J = 16.8, 5.9 Hz, 1H), 3.60 (tdq, J = 16.4, 11.0, 5.5 Hz, 6H), 3.23 – 3.03 (m, 4H), 2.95 (td, J = 14.2, 8.3 Hz, 2H), 2.83 (dd, J = 14.1, 4.7 Hz, 1H), 2.64 (dd, J = 14.2, 9.1 Hz, 1H), 2.23 (dq, J = 16.5, 7.0 Hz, 4H), 2.05 (dt, J = 13.7, 6.7 Hz, 1H), 1.89 (t, J = 10.5 Hz, 2H), 1.68 (dh, J = 13.3, 7.6, 6.3 Hz, 2H), 1.55 – 1.43 (m, 5H), 1.04 (d, J = 6.2 Hz, 3H), 0.97 – 0.84 (m, 11H).

^13^C NMR (101 MHz, DMSO*-d_6_*) δ 174.63, 173.75, 172.29, 171.94, 171.75, 171.35, 171.10, 170.64, 170.27, 168.97, 168.23, 157.21, 156.20, 136.52, 136.46, 130.47, 128.09, 127.85, 127.78, 124.08, 123.97, 121.31, 118.73, 115.35, 110.57, 110.33, 66.95, 64.18, 62.19, 58.55 – 58.16 (m), 57.76, 55.44 (d, J = 3.1 Hz), 53.85, 52.30, 51.63, 42.25, 41.51, 30.61, 30.37, 28.09, 24.45, 23.48, 22.13, 19.79, 18.84, 18.01.

**F)** **FITC-Ahx-VLGREEWSTSYW**

^1^H NMR (400 MHz, DMSO*-d_6_*) δ 10.90 – 10.55 (m, 2H), 10.04 (d, J = 67.4 Hz, 2H), 9.12 (s, 0H), 8.31 – 8.12 (m, 2H), 8.12 – 7.66 (m, 10H), 7.58 (t, J = 7.9 Hz, 2H), 7.42 (t, J = 5.8 Hz, 1H), 7.31 (t, J = 7.5 Hz, 2H), 7.22 – 7.10 (m, 3H), 7.10 – 6.81 (m, 6H), 6.76 – 6.47 (m, 7H), 4.95 (s, 0H), 4.61 (q, J = 7.4 Hz, 1H), 4.49 – 3.90 (m, 20H), 3.79 (dd, J = 16.6, 5.6 Hz, 1H), 3.69 – 3.37 (m, 3H), 3.23 – 3.01 (m, 4H), 3.01 – 2.87 (m, 2H), 2.87 – 2.77 (m, 1H), 2.63 (dd, J = 14.1, 9.4 Hz, 1H), 2.36 – 2.06 (m, 5H), 2.04 – 1.81 (m, 2H), 1.81 – 1.38 (m, 11H), 1.39 – 1.17 (m, 3H), 1.03 (d, J = 6.3 Hz, 3H), 0.91 – 0.79 (m, 11H).

^13^C NMR (101 MHz, DMSO*-d_6_*) δ 174.54, 174.18 – 173.25 (m), 172.82, 171.93, 171.74, 171.68, 171.34, 171.08, 170.63, 170.26, 169.01, 159.96, 157.12, 156.19, 152.36, 136.46, 130.46, 129.47, 128.10, 127.78, 124.02, 121.27, 118.70, 115.35, 113.05, 110.57, 110.32, 110.22, 102.71, 62.16, 58.30, 55.43, 53.84, 52.39 (d, J = 11.4 Hz), 51.57, 44.26, 42.43, 36.98, 36.77, 35.57, 30.67, 28.61, 28.11, 26.57, 25.65, 25.29, 24.55, 23.47, 22.02, 19.76, 18.74.

**Figure S9. ^1^H and ^13^C NMR spectra of peptides: A) FALGEA, B) FITC-Ahx-FALGEA, C) YHWYGYTPENVI, D) FITC-Ahx-YHWYGYTPENVI, and E) VLGREEWSTSYW, F) FITC-Ahx-VLGREEWSTSYW.** NMR were performed on a 400 MHz spectrometer (Bruker) Nanobay console with broadband BBO probe with Z shim gradient and ATMA. 15 mg of samples are diluted in 750 µL in DMSO-d_6_. For ^1^H experiments: Number of scans NS = 64, Number of dummy scans DS = 2, Size of FID TD = 65k, Relaxation time D1 = 8 sec. For ^13^C experiments: Number of scans NS = 1024 for A) & B), 3036 for the other peptides, Number of dummy scans DS = 4, Size of FID TD = 65k, Relaxation time D1 = 2 sec.

.

**A) FALGEA**


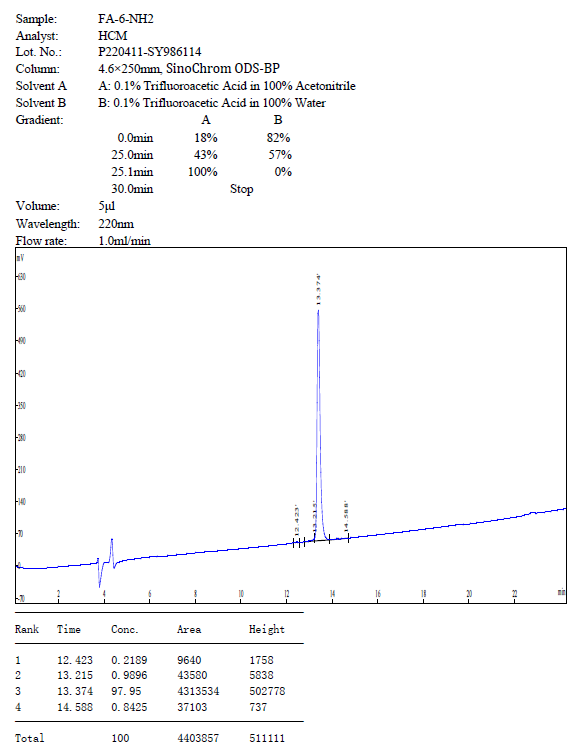


**B) FITC-Ahx-FALGEA**

**C) YHWYGYTPENVI**

**D) FITC-Ahx-YHWYGYTPENVI**

**E) VLGREEWSTSYW**

**F) FITC-Ahx-VLGREEWSTSYW**

**Figure S10. HPLC chromatograms of the peptides: A) FALGEA, B) FITC-Ahx-FALGEA, C) YHWYGYTPENVI, D) FITC-Ahx-YHWYGYTPENVI, E) VLGREEWSTSYW, and F) FITC-Ahx-VLGREEWSTSYW**

**Table S4. Molar mass from MS and purity from HPLC of FITC-Ahx-peptides.**

| **Peptide** | **M**  **(g/mol)** | **Purity** |
| --- | --- | --- |
| **FITC-Ahx-FALGEA-CONH_2_** | 1108.22 | 95.66% |
| **FITC-Ahx-YHWYGYTPENVI-CONH_2_** | 2043.21 | 98.07% |
| **FITC-Ahx-VLGREEWSTSYW-CONH_2_** | 2014.17 | 97.11% |


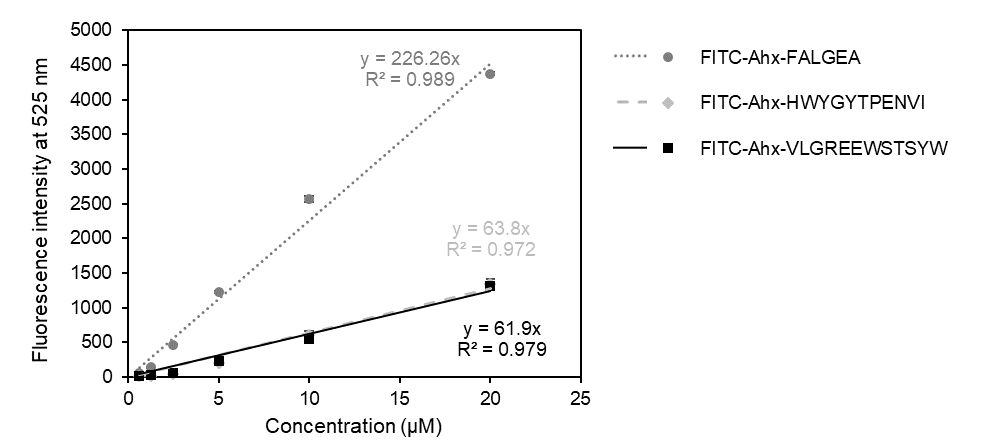


**Figure S11. Fluorescence intensity at 525 nm versus FITC-Ahx-peptide molar concentration.** Data shown for FITC-Ahx-FALGEA (circle, dark gray), FITC-Ahx-YHWYGYTPENVI (diamond, light gray), and FITC-Ahx-VLGREEWSTSYW (square, black). Slopes of the linear regressions were used to determine correction factors to normalize MFI obtained by flow cytometry.

**Table S5. Slope, R^2^ and correction factor for each peptide.**

| **Peptide** | **FITC-Ahx-FALGEA** | **FITC-Ahx-YHWYGYTPENVI** | **FITC-Ahx-VLGREEWSTSYW** | **Correction to** |
| --- | --- | --- | --- | --- |
| Slope | 226.3 | 63.8 | 61.9 | 100.0 |
| R² | 0.989 | 0.972 | 0.979 |  |
| Correction Factor | 2.26 | 0.64 | 0.62 | 1.00 |
